# Development and Application of Automatized Routines for Optical Analysis of Synaptic Activity Evoked by Chemical and Electrical Stimulation

**DOI:** 10.3389/fbinf.2022.814081

**Published:** 2022-02-15

**Authors:** Debarpan Guhathakurta, Enes Yağız Akdaş, Anna Fejtová, Eva-Maria Weiss

**Affiliations:** Department of Psychiatry and Psychotherapy, Universitätsklinikum Erlangen, Friedrich-Alexander Universität Erlangen-Nürnberg, Erlangen, Germany

**Keywords:** segmentation algorithm, synapse detection, synaptic vesicle recycling, electrical stimulation, chemical depolarization, cultured neurons, image processing

## Abstract

The recent development of cellular imaging techniques and the application of genetically encoded sensors of neuronal activity led to significant methodological progress in neurobiological studies. These methods often result in complex and large data sets consisting of image stacks or sets of multichannel fluorescent images. The detection of synapses, visualized by fluorescence labeling, is one major challenge in the analysis of these datasets, due to variations in synapse shape, size, and fluorescence intensity across the images. For their detection, most labs use manual or semi-manual techniques that are time-consuming and error-prone. We developed SynEdgeWs, a MATLAB-based segmentation algorithm that combines the application of an edge filter, morphological operators, and marker-controlled watershed segmentation. SynEdgeWs does not need training data and works with low user intervention. It was superior to methods based on cutoff thresholds and local maximum guided approaches in a realistic set of data. We implemented SynEdgeWs in two automatized routines that allow accurate, direct, and unbiased identification of fluorescently labeled synaptic puncta and their consecutive analysis. SynEval routine enables the analysis of three-channel images, and ImgSegRout routine processes image stacks. We tested the feasibility of ImgSegRout on a realistic live-cell imaging data set from experiments designed to monitor neurotransmitter release using synaptic phluorins. Finally, we applied SynEval to compare synaptic vesicle recycling evoked by electrical field stimulation and chemical depolarization in dissociated cortical cultures. Our data indicate that while the proportion of active synapses does not differ between stimulation modes, significantly more vesicles are mobilized upon chemical depolarization.

## Introduction

Neurotransmission is crucial for brain development, cognition, learning, and memory processes. In neuronal synapses, neurotransmitters are stored in synaptic vesicles (SVs). Upon stimulation of neurons, these vesicles fuse with the presynaptic plasma membrane to release neurotransmitter into the synaptic cleft, which is the key step in synaptic transmission. To preserve the presynaptic structure and to ensure effective vesicular release during repetitive stimulations, SVs are retrieved from the presynaptic membrane and subsequently refilled with neurotransmitters. To study their properties, synapses in neurons can be visualized as synaptic puncta in neurons *in vitro*, *ex vivo*, or *in vivo* with fluorescence microscopy utilizing antibodies against pre- and postsynaptic proteins (Ivanova et al., 2020; Anni et al., 2021) or using genetically encoded reporter constructs (Ng et al., 2002; Welzel et al., 2011). Reliable detection of synaptic puncta is crucial for proper quantification of synaptic properties. In the past, automatized segmentation algorithms emerged as tools to reduce time need and human bias (Ippolito and Eroglu, 2010; Danielson and Lee, 2015; Kulikov et al., 2019). Nowadays, sophisticated segmentation algorithms based on machine learning are able to segment synapses precisely and comprise approaches working with very small sets of training data (Berg et al., 2019; Stringer et al., 2021). However, downstream postprocessing of bulk images and merging of received data are difficult. In fact, most labs still rely on human experts carrying out detection of synaptic puncta manually or semi-manually (Abraira et al., 2017; Ippolito and Eroglu, 2010; O'Neil et al., 2021). This procedure is time-consuming and error-prone and relies on reduced data amount. We think that routines, enabling a full analysis that includes preprocessing steps and postprocessing calculations, can improve this. Hence, we developed the segmentation algorithm SynEdgeWs that we implemented in frameworks to realize fully automatized routines performing image preprocessing, precise and robust puncta segmentation, and postprocessing of data. SynEval routine allows the analysis of three-channel images and embeds the readout of synaptic puncta features such as number, fraction, and emitted mean fluorescence intensity (MFI). ImgSegRout routine processes image stacks such as time-lapse imaging sequences. We applied ImgSegRout on a realistic live-cell imaging data set from experiments where SV release was monitored using genetically encoded markers, the so-called synaptic phluorins (Royle et al., 2008). Finally, as a proof of concept, we tested SynEval routine on a realistic data set intended to compare different approaches to induce neurotransmitter release in cultured neurons, namely electrical stimulation via field electrodes and chemical depolarization.

## Methods

### Preprocessing

Efficient preprocessing of images is crucial for proper segmentation of synaptic puncta. In the first step, convolution of the original image creates a background image that is subtracted from the original image afterward (Supplementary Methods S1.1) (Sternberg, 1983). Negative values are set to zero and linear normalization enhances the contrast of acquired images. The preprocessing routine is additionally equipped with a retouching function for very bright regions that may disturb proper segmentation. This is an optional function, selectable via graphical user interface (GUI). Thereby, based on its characteristic bimodal shape, intensity histogram of the original image enables determining of a cutoff threshold value in-between the maxima to outline bright regions (Supplementary Methods S1.1, Supplementary Figure S1). Subsequent dilation and flood filling were implemented with MATLAB built-in functions. The resulting binary image masks the original image and the values of pixels within the mask are replaced with the corresponding pixel values from the background image. Subsequently, the background is subtracted from the whole image.

### Segmentation Algorithm SynEdgeWs

**FIGURE 1:**
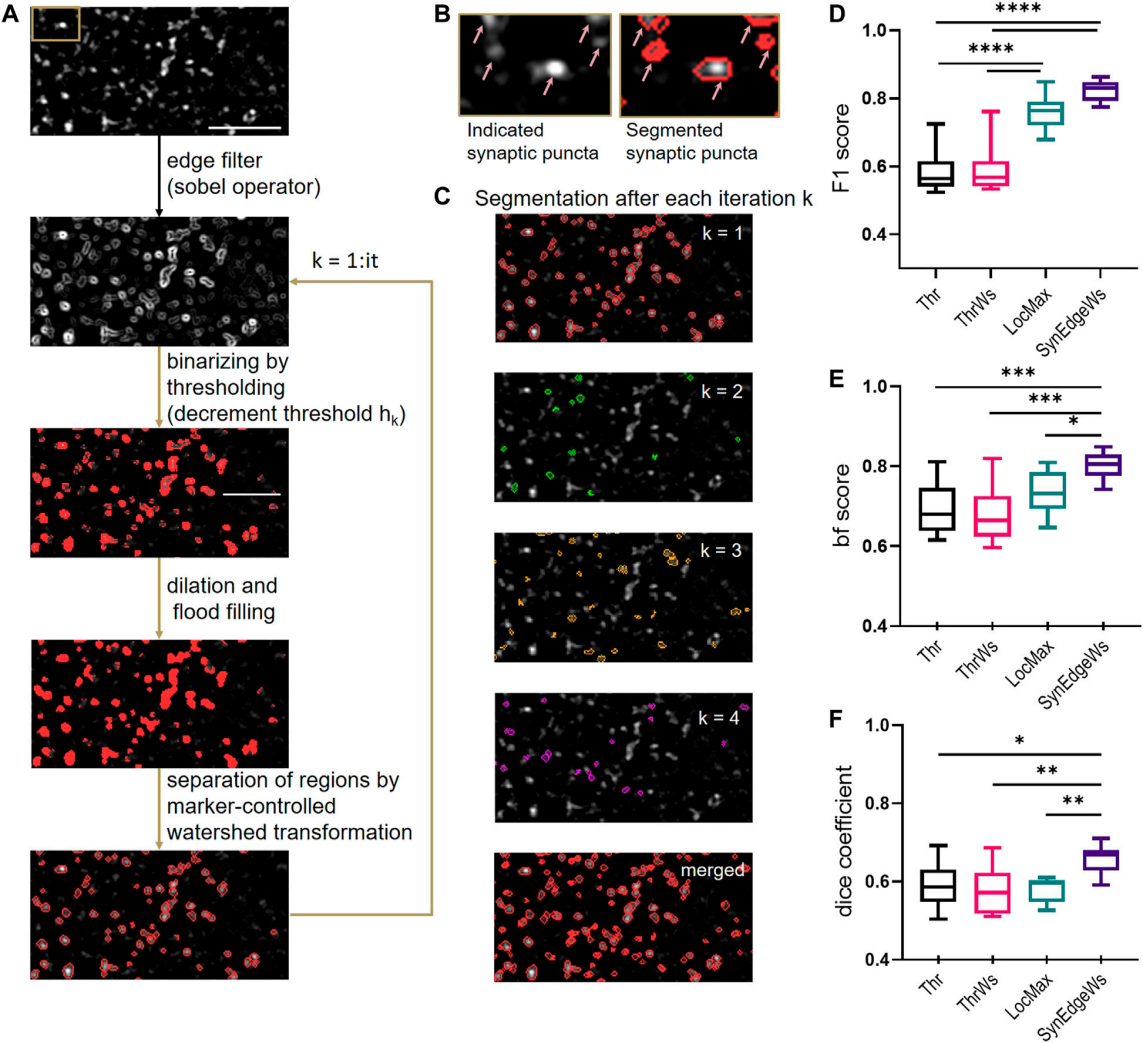
Design and benchmarking of SynEdgeWs segmentation algorithm. **(A)** Illustration of puncta detection procedure by iterative gradient threshold application in SynEdgeWs exemplified on synapsin marker staining. The upper image is the initial image (synaptic staining of cultured neurons). SynEdgeWs applies an edge filter (sobel operator) and iteratively performs a rough segmentation by gradient threshold refined by followed dilation/flood filling operation and marker controlled watershed transformation. **(B)** Close-up (section indicated in A, upper image) of synaptic puncta marked by arrows (left) and their segmentation (right). **(C)** Visualization of detected ROIs after each iteration (*n* = 4, step 1–4) on the initial image. The last row shows merged segmentation mask after four iterations on the initial image. **(D–F)** Performance of SynEdgeWs (with iteration number it = 2) was benchmarked against thresholding (Thr), thresholding with subsequent marker-controlled WS (ThrWs), and detection of local maxima controlled by global threshold (LocMax) using cropped images stained against synapsin (*n* = 10). Validation of SynEdgeWs by F1 score **(D)**, bf score **(E)**, and dice coefficient **(F)**. For statistics one-way ANOVA with multiple comparisons by Tukey was done: ****p < 0.0001, ***p < 0.001, **p < 0.01, *p < 0.05. The box indicates the interquartile distance with median, and the whiskers are plotted in minimum to maximum range.

We developed SynEdgeWs to detect automatically fluorescently labeled synaptic puncta without user intervention (detailed flowchart in Supplementary Material Figure S2, Figure 1A). While customized to work within the presented routines, SynEdgeWs implementation in new or modified routines is easy. In brief, an edge filter using sobel operator (Kanopoulos et al., 1988) calculates the image gradient (Figure 1B). Determined on an image gradient histogram, the application of the gradient threshold outlines the edges of synaptic puncta as a rough segmentation that is followed by dilation and flood-filling operations. To separate potentially connected puncta, marker-controlled watershed transformation operates within each section originating from intensity centroids. Afterward morphological operators (dilation/erosion) discard potential artifacts. To refine contour of regions of interest (ROI), thresholding checks border pixel values. Regions with a size beyond a certain range are discarded. Therefore, in the frameworks, minimum and maximum pixel numbers are calculated from expected synaptic puncta size in micrometer, camera pixel size, magnification, and binning adjustable via the GUI. The algorithm works with an iteratively decreasing image gradient threshold to overcome heterogeneous fluorescence intensity emitted by puncta (Figure 1C, Supplementary Material—Methods S1.2). For each iteration, the coordinates of detected synaptic puncta were stored in order to merge them finally. ROI detected during one iteration was excluded for the following iterations. This procedure avoids the detection of large regions that would be difficult to separate consecutively by watershed transformation. The user can determine the number of iterations *via* the GUI.

### Routine SynEval for Segmentation of Antibody-Stained Synapses in a Multichannel Approach

**FIGURE 2:**
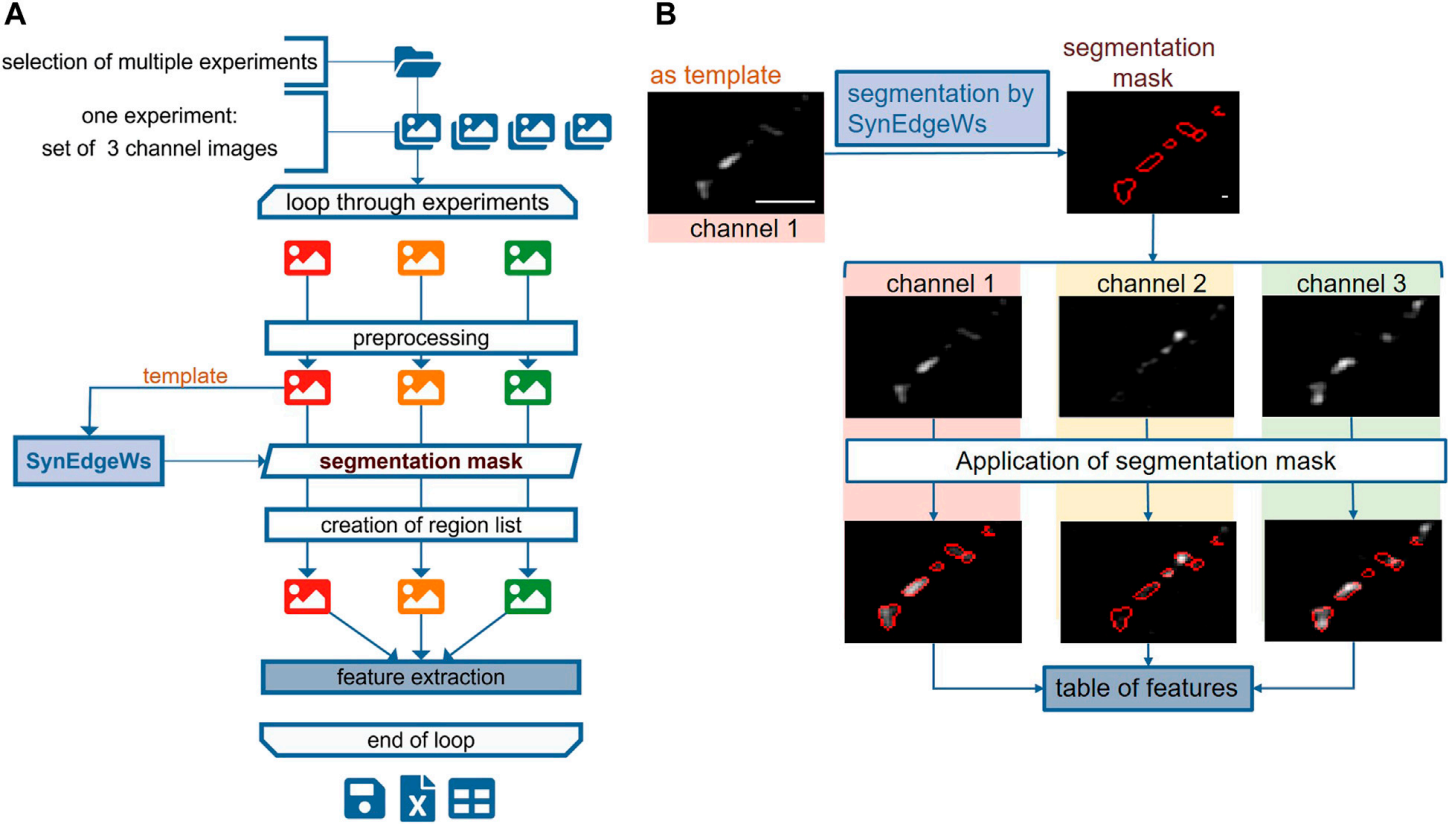
SynEval for detection and analysis of synapses in three-channel immunofluorescent images. The schema in **(A)** shows all steps of SynEval routine. **(B)** Image recorded in channel 1 (here Syn1,2 staining) serves as a template to create segmentation mask. The segmentation mask is applied on images from channels 2 (Syt1Ab uptake) and 3 (anti-VGAT staining) to read out parameters and create table of features. Scale bar is 4 µm.

The routine SynEval analyzes three-channel data in a batch process (Figures 2A,B). A GUI enables selecting images as TIFF files for each channel and configuring settings for the determination of the valid synaptic puncta size range in pixel counts (Supplementary Material Table S1). All images undergo preprocessing. The image recorded in channel 1 is set as a template. A segmentation mask and the corresponding list of ROI coordinates arise from running SynEdgeWs on this template. The ROI coordinates are transferred to channel 2 and 3 images and the MFI of each ROI from all the channels is obtained. To evaluate signal colocalization, the program determines a threshold for the signal in channels 2 and 3. Therefore, the application of edge filter, dilation, and flood filling results in a rough segmentation. Within this segmented region, the median of the lowest 1% fluorescence intensity is calculated and defines the threshold. Further postprocessing calculations provide a feature table (Supplementary Material Table S2), which is exported as an MS excel file.

### Routine ImgSegRout for Monitoring Fluorescence Signals Derived From Puncta

**FIGURE 3:**
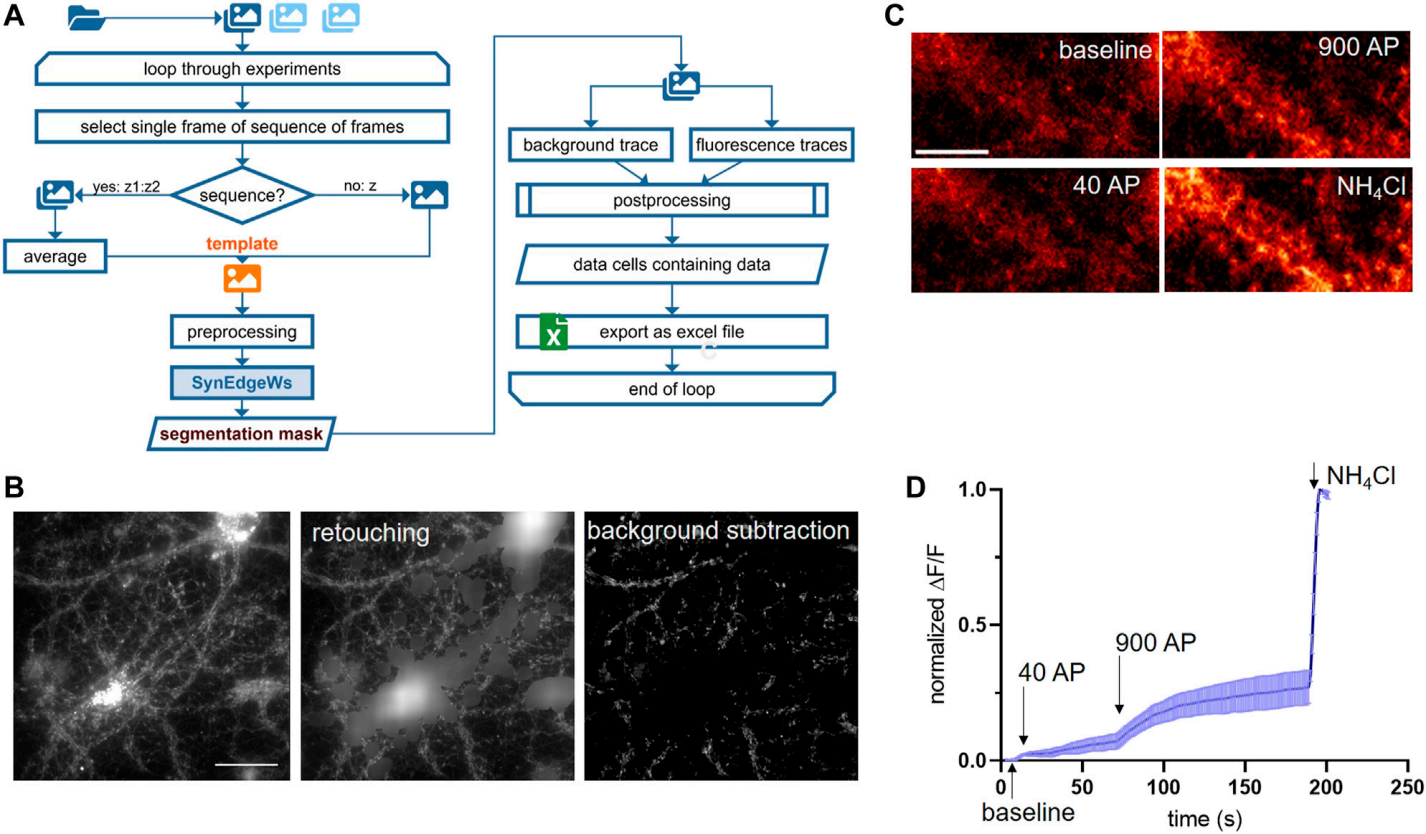
ImgSegRout for detection and analysis of synapses in image stacks. **(A)** Flowchart in A depicts all steps of ImgSegRout. **(B)** Example of preprocessing of images during ImgSegRout. Here, images of neuronal cultures expressing SypmOr sensor for monitoring of SV recycling were processed. Shown are the raw image (left), image after retouching procedure (middle), and image after background subtraction (right). Scale bar is 40 µm. **(C)** Illustration of neurons from imaging experiment to monitor SV release. Shown are cells upon stimulation with 40 AP (20 HZ) and 900 AP (20 Hz), respectively, and the application of NH_4_Cl to alkalize SV and to visualize the total amount of SV. Scale bar is 15 µm. **(D)** Representative mean curve derived from three experiments. The data points represent the mean values of normalized MFI traces (ΔF/F) with plotted error (SEM). In an experiment, normalized MFI values were calculated by averaging all traces derived from individual ROIs and by normalizing in the range of NH_4_Cl signal and baseline (F = F_NH4Cl_—F_base_; ΔF = F_frame_—F_base_).

ImgSegRout processes time-lapse recordings saved as image stacks. It works in a batch mode and allows the operator to select several image stack files at once (Figure 3A). The routine is based on our routine described in Anni et al. (2021) modified by herein-introduced segmentation algorithm SynEdgeWs and preprocessing procedure (process flow in Figure 3A). In brief, similar to SynEval, a GUI prompts to adjust settings for puncta size calculation, to select preprocessing features such as retouching and to insert the iteration number using SynEdgeWs (Supplementary Material Table S1). Moreover, postprocessing steps such as bleaching correction are selectable. Additionally, either by selecting a single frame or by selecting a sequence of frames consecutively averaged, the user determines a template for the segmentation process in SynEdgeWs. Subsequently, a multiple TIFF file is loaded. SynEdgeWs detects ROI on the template and returns a list of coordinates. ROI coordinates are transferred to each frame of the whole stack and MFI is read out. Additionally, the read-out process returns a background trace containing one background value per frame. Postprocessing includes subtraction of background values from individual fluorescent signal traces as well as smoothing and optional bleaching correction described in Anni et al. (2021). ImgSegRout exports all results as a MS excel file.

### Primary Neuronal Cultures

Dissociated primary rat neuronal cultures were prepared exactly as described previously (Anni et al., 2021). The experiments involving animals in this study were approved by local animal welfare officer (FAU: TS12/2016 and TS13/2016), in accordance with the European Directive 2010/63/EU and German animal welfare law. Briefly, cortices from E18 rat embryo were collected and cell suspension was obtained after trypsinizatin and mechanical trituration. Cells were plated in DMEM containing 10% (v:v) fetal calf serum, L-glutamine, and antibiotics on poly-L-lysine coated 18 mm Menzel glass coverslips at density of 120,000 cells/ml and kept at 37°C in 5% CO2 atmosphere. 1 h later media was replaced to Neurobasal growth medium supplemented with B27, L-Glutamine, and antibiotics. Neurons were grown for 18–21 days *in vitro* (DIV) prior to all experiments (Supplementary Material Table S3).

### Immunocytochemistry and Synaptotagmin1 Antibody Uptake Assay

Synaptotagmin1 antibody (Syt1Ab) uptake assay was carried out using Syt1Ab as described previously with slight modifications (Anni et al., 2021). For chemical stimulation, high KCl-Tyrode’s buffer (TB) containing in mM: 69 NaCl, 50 KCl, 2 CaCl_2_, 2 MgCl_2_, 30 glucose, 25 HEPES, pH 7.4 and Syt1Ab (1:250 dilution) was applied to coverslips with DIV 18–21 neurons for 4 min at room temperature (RT). Thereafter, neurons were shortly washed and fixed in 4% (w:v) paraformaldehyde. For electrical stimulation, neurons were placed in a stimulation chamber and immersed in physiological TB, containing in mM: 119 NaCl, 2.5 KCl, 2 CaCl_2_, 2 MgCl_2_, 30 glucose, 25 HEPES, pH 7.4 and Syt1Ab. A train of 900 pulses (90 mA, 1 ms each) was delivered at 20 Hz using submersed electrodes. After 1 min, neurons were shortly washed and fixed in 4% (w:v) paraformaldehyde. The following steps were identical for electrically and chemically stimulated samples. For blocking and permeabilization coverslips were incubated in 10% (v:v) FCS, 0.1% (w:v) glycine, and 0.3% (v:v) TritonX 100 in PBS for 40 min. Primary antibody against VGLUT1 (1:1,000), VGAT (1:1,000), and synapsin 1,2 were applied overnight at 4°C in 1:1,000 dilution. The fluorescently labeled secondary antibodies were applied for 1 h at RT. All antibodies were diluted in PBS containing 3% (v:v) FCS. Coverslips were mounted in Mowiol. Images of immunofluorescence for all channels were acquired exactly as described previously (Anni et al., 2021) (Supplementary Material Table S3).

### Preparation of Lentiviral Construct

To express the pH-sensitive synaptophysin–mOrange [SypmOr (Egashira et al., 2015)] in neuronal cultures, the SypmOr sequence was cloned into a FULW lentiviral vector (i.e., FUW with a modified multiple cloning site) using NEBuilder^®^ HiFi DNA Assembly (NEB) through EcoRI and BamHI restriction sites. The production of virus in HEK293T cells was done exactly as described in Anni et al. (2021). To transduce neurons, 100 µL of lentivirus containing medium was applied per coverslip at DIV 2 (Supplementary Material Table S3).

### Live Imaging of SV Recycling Using SypmOr

Imaging was performed as in Anni et al. (2021) with minor modifications. Coverslips with neurons (DIV18-21) were placed in an electrical field stimulation chamber and imaged at RT in physiological TB containing 10 µM CNQX, 50 µM APV, pH 7.4, and 1 µM bafilomycin A1 on an epifluorescence microscope, using an automated perfect focus system (PFS) and 60X/NA1.2 water-immersion objective. Stimulus was generated using A 385 stimulus isolator connected to STG-4008 stimulus generator (Multi Channel Systems, Reutlingen, Germany). Subsequent to stimulations, TB containing 60 mM NH_4_Cl was applied to achieve alkalization across all membranes. SypmOr fluorescent dye was excited at 543/22 with a Led-HUB lamp and time-lapse images were acquired using a Cy3 filter (emitter 593/40) at the frequency of 1 Hz using iXon EM + 885 EMCCD Andor camera controlled by VisiView software in 2 * 2 binning mode. Data were exported as stack files (.stk) containing frames with 502 × 501 pixels of 16-bit monochromatic intensity values (Supplementary Material Table S3, S4). For further processing, stack files are converted into multiple TIFF files.

## Results

### Performance of Segmentation and Routines

The aim of both presented routines is the fast, unbiased, and reproducible identification of synaptic puncta from images obtained by fluorescence microscopy and consecutive calculation returning a table of results as an Excel file. The in-house developed segmentation tool SynEdgeWs is the essential core algorithm of both routines and is imbedded in a framework of pre- and postprocessing procedures to allow direct usage on data with the purpose of saving time and reducing error potential.

#### Benchmarking of Segmentation Tool SynEdgeWs

Binarization of images by automatically determined cutoff threshold (Thr) (Sezgin and Sankur, 2004; Glebov, 2019) and local maxima determination, controlled by global threshold (LocMax) (Sbalzarini and Koumoutsakos, 2005; Xu et al., 2011) are still commonly used methods for image segmentation that run without user intervention and training data render them capable to run within the presented routines. To test SynEdgeWs algorithm, we benchmarked its performance against these methods by implementing them into the same environment in MATLAB. Since watershed transformation is a common method to separate connected puncta, we additionally implemented that to Thr (ThrWs) (Richter et al., 2018; Guo et al., 2019) (Supplementary Material Methods S1.3). To generate a reference segmentation as ground truth (ROI_ref_), a human expert carried out manual segmentation of synaptic puncta on ten cropped images using Image Segmenter App (MATLAB). The same images were subsequently segmented by the four automatic segmentation methods resulting in respective ROI_auto_. To compare all tested algorithms, F1 score was calculated. F1 is an established parameter to benchmark accuracy calculated as the harmonic mean of the performance metrics precision (positive predictive value) and recall (sensitivity) (Dice, 1945; Sørensen, 1948; Fawcett, 2006). Here, the calculation of F1 score underlies the comparison of individual ROIs (Supplementary Material Method S1.4). Additionally, we used built-in functions in MATLAB to measure further parameters to quantify segmentation quality. These are the F1 score, which compares the binary segmentation masks at pixel level, hereinafter referred to as dice coefficient (dice) (The MathWorks, 2017a) and the contour-matching score, also called boundary F1 score (bf score) (Csurka et al., 2004; The MathWorks, 2017b).

Benchmarking SynEdgeWs against LocMax yielded in significantly higher values for the measure dice (SynEdgeWs: 0.658 ± 0.011, LocMax: 0.580 ± 0.010, *p* = 0.0040) as well as bf score (SynEdgeWs: 0.802 ± 0.011, LocMax: 0.733 ± 0.016, *p* = 0.0461) and higher values for F1 score (SynEdgeWs: 0.822 ± 0.010, LocMax: 0.761 ± 0.016). For all measures, SynEdgeWs significantly outperforms Thr (F1 score: 0.582 ± 0.019 *p* < 0.0001; bf score: 0.693 ± 0.021, *p* = 0.0007; dice: 0.591 ± 0.018, *p* = 0.0158) and ThrWs (F1 score: 0.590 ± 0.022, *p* < 0.0001; bf score: 0.681 ± 0.021, *p* = 0.002; dice: 0.577 ± 0.018, *p* = 0.0025) in all measures (Figures 1D–F).

#### SynEval for Detection and Analysis of Synapses in Three-Channel Immunofluorescence Images

**FIGURE 4:**
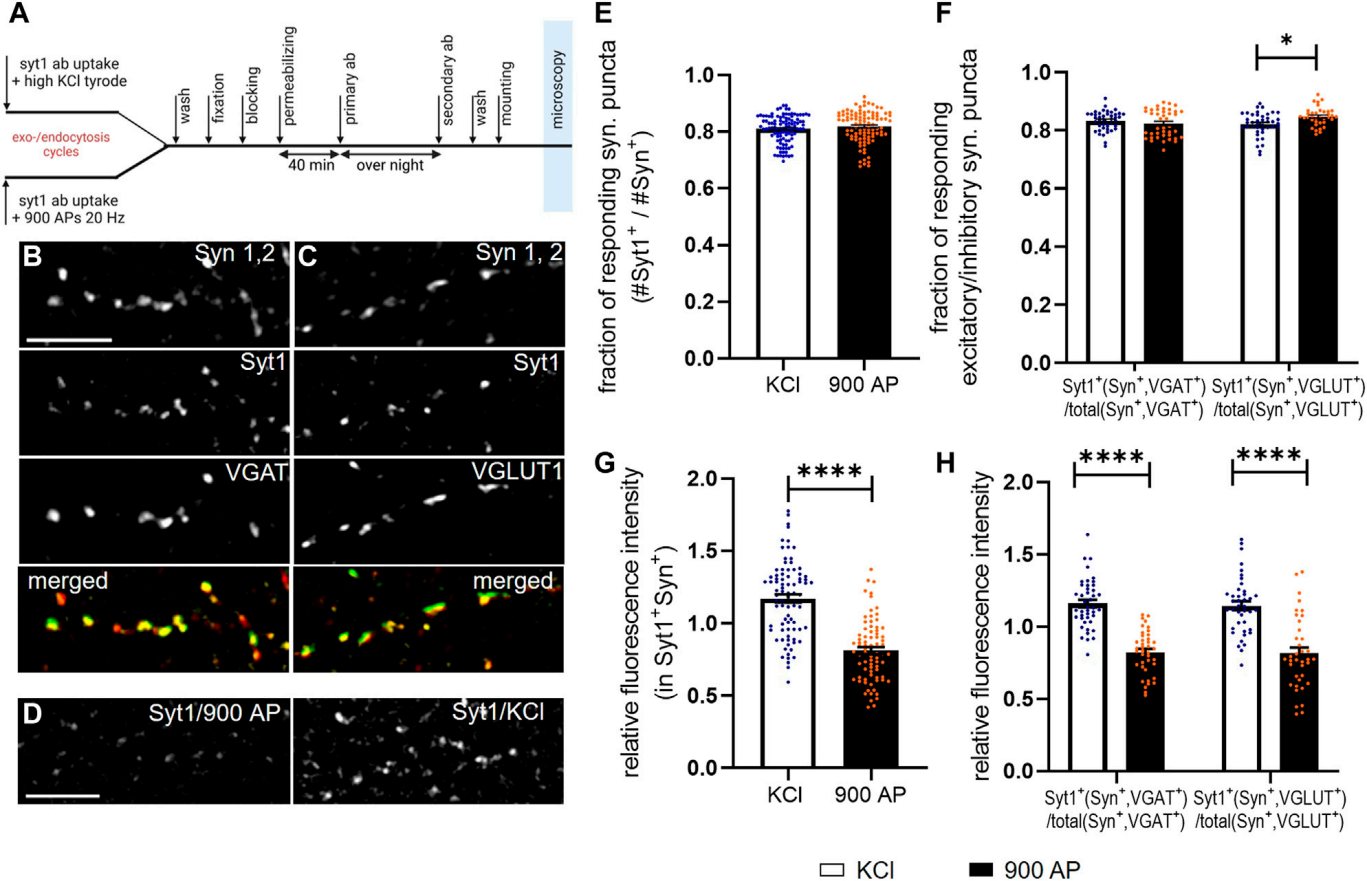
Application of SynEval for the analysis of SV recycling evoked by electrical and chemical stimulation. Experimental design of Syt1Ab uptake with consecutive immunostaining is depicted in **(A)**. **(B–C)** The representative pictures are shown for Syn 1,2 staining used to determine synaptic puncta, Syt1Ab uptake assay upon chemical depolarization (50 mM KCl, 4 min) and staining for VGAT for inhibitory synapses **(B)** and VGLUT1 for excitatory synapses **(C)**, and respective merged images. **(D)** The representative images of Syt1 uptake upon electrical stimulation (900 AP/20 Hz) and chemical depolarization. Scale bars are 10 µm. **(E,F)** Quantification of active synapses evaluated as fraction of Syt1-positive puncta (Syt1^+^) out of all synapsin-positive puncta **(E)** or on inhibitory or excitatory synapses defined by VGAT or VGLUT1 staining **(F)** upon chemical depolarization (KCl) and electrical stimulation (900 AP) stimulation. N was 84 (KCl) or 77 (900 AP) visual fields derived from eight coverslips for each group in E and 43 (KCl/VGAT), 40 (900 AP/VGAT), 40 (KCl/VGLUT1), 35 (900 AP/VGLUT1) visual fields derived from four coverslips per group in F. **(G)** MFI of active (i.e., double positives Syt^+^Syn^+^) synapses upon chemical depolarization or electrical stimulation. **(H)** Same analysis as in G but inhibitory (VGAT^+^) and excitatory (VGLUT1^+^) synapses were identified by staining and evaluated separately. N numbers were same as in E and F, in the graph bars depict the mean values and whiskers correspond to SEM. Significance was assessed using Student’s t-test, significance is depicted as ****p < 0.0001, *p < 0.05.

The MATLAB-based routine SynEval facilitates analysis of three-channel recordings in a batch process. The image recorded in channel 1 is set as a template to create a segmentation mask and a list of coordinates of detected ROI (Figures 2A,B). The ROI coordinates are transferred to the images recorded in channels 2 and 3 to read out parameters (Supplementary Material Table S2). To test SynEval on a realistic dataset, probes immunostained against synapsin 1,2 (Syn 1,2), a synaptic marker, were recorded in channel 1. The signal in channel 2 corresponded to Syt1 antibody labeling. This labeling had been previously performed in living cells to mark active synapses undergoing neurotransmitter release during antibody incubation (Kraszewski et al., 1995). The signal in channel 3 corresponded to staining for vesicular glutamate transporter 1 (VGLUT1), a marker for excitatory synapses (Figure 4B). Compared with manual analysis, this routine enables faster analysis. We tested running time (*n* = 150 images split into 15 runs) of our routine by using a built-in function stopwatch timer by MATLAB resulting in a mean value of 39.1 ± 4.8 s (Supplementary Material Table S5). Analysis of the same data, performed by a skilled experimenter using an optimized Fiji plugin (Wang et al., 2020), needed about 240 s per experiment. Additional 240 s were needed for postprocessing data carried out in MS Excel (Supplementary Material Methods S1.5). Thus, the time advantage gained by SynEval is around one order of magnitude compared with the semi-manual method. Time requirement for the user is further reduced courtesy of the batch mode.

#### ImgSegRout for Detection and Analysis of Synapses in Image Stacks

Time-lapse fluorescence imaging of optical probes targeted toward the lumen of SVs (synapto-pHluorins) is a common method to investigate release and recycling of SVs at the level of individual synapses, which is a proxy for neurotransmission (Sankaranarayanan et al., 2000). We developed ImgSegRout to monitor synapto-pHluorin fluorescence signals from time-lapse recordings, but, in general, the routine is capable of extracting fluorescence traces derived from any fluorescent puncta recorded as an image stack. It processes data in a batch mode (Figure 3A). To test ImgSegRout, we generated a realistic dataset by live-imaging neurons expressing the SypmOr reporter for monitoring SV fusion and retrieval (Egashira et al., 2015). In this case, we implemented an approach described earlier by Burrone and colleagues (Burrone et al., 2006). Specifically, imaging was performed in the presence of bafilomycin to prevent vesicle reacidification, which allows visualization of cumulative release of SVs of different physiological properties. Release of a readily releasable pool of vesicles was induced by electrical field stimulation with 40 APs (pulses) at 20 Hz, release of all releasable vesicles was achieved by delivery of 900 APs at 20 Hz (Figures 3C,D). In these experiments, expression of SypmOr reporter resulted in a strong fluorescence signal in neuronal cell bodies, which hampered the reliable segmentation process. Therefore, we applied a function integrated in preprocessing to retouch these very bright areas and to render images suitable for the segmentation process (Figure 3B). Testing performance of ImgSegRout by analyzing several real data experiments (*n* = 12 split in 3 runs) using the built-in stopwatch time function in MATLAB yielded in an averaged running time of 29.99 s per image stack with 260 images, 502 × 501 pixels. We switched off bleaching correction, because the bleaching was minimal in these experiments.

### Application of SynEval to Compare SV Recycling Induced by Chemical or Electrical Stimulation

Finally, we employed SynEval on realistic data with the aim of comparing SV release induced by chemical depolarization and electrical field stimulation. These two methods are broadly used in the field, but direct comparison of the data obtained by these alternative approaches was not yet performed. To close this gap, we labeled recycling vesicles evoked 1) by brief chemical depolarization with 50 mM KCl or 2) by electrical field stimulation with 900 APs at 20 Hz applied via submerged parallel field electrodes (Figure 4A). We used an antibody against luminal domain of SV protein Syt1 (Syt1Ab). This antibody binds its epitope only upon fusion to SV with plasma membrane (i.e., during depolarization/stimulation), internalized during compensatory endocytosis and thus labels vesicles that have underwent exo- and endocytosis cycle during time of experiment. Following stimulation, cells were fixed and processed for immunostaining with antibodies for presynaptic marker Syn 1,2, as well as for marker of inhibitory (vesicular GABA transporter, VGAT) (Figure 4B) or excitatory (VGLUT1) (Figure 4C) synapses. Images were analyzed, using SynEval routine (Figure 2A). Syn 1,2 staining had been recorded in channel 1 to create segmentation mask with SynEdgeWs (Figure 2B). The segmentation mask determined ROIs on images from channel 2 (Syt1Ab uptake) and channel 3 (VGAT and VGLUT1, respectively) and application of threshold identified ROIs as positive for respective marker. To compare stimulation methods, proportion of synapses positive for Syt1Ab uptake as well as MFI of Syt1Ab uptake signal were analyzed (Figures 4F–H). While the first parameter reveals proportion of presynaptically silent synapses, the second relates to the relative number of SVs, which underwent exocytosis upon the respective stimulation at individual synapses and is a good proxy for presynaptic efficacy. The overall number of active (i.e., responding) synapses in relation to the total amount of synapses was similar upon both types of stimulation (Figure 4E, KCl: 0.829 ± 0.004; AP 900: 0.0.835 ± 0.005). In the next step, we analyzed proportion of active inhibitory and excitatory synapses. No difference was obvious in the proportion of inhibitory synapses, minor but significant increase was detected in the proportion of excitatory synapses upon electrical stimulation (Figure 4F, VGLUT1^+^, KCl: 0.813 ± 0.007; AP 900: 0.844 ± 0.007/VGAT^+^, KCl: 0.935 ± 0.003; AP 900: 0.919 ± 0.004). In contrast, analyzing FI of Syt1Ab, depicted as relative FI related to overall mean, showed increased labeling upon depolarization with KCl compared with electrical stimulation (Figure 4G, KCl: 1.172 ± 0.028; AP 900: 0.8118 ± 0.024). This was true for both inhibitory and excitatory synapses (Figure 4H, VGLUT1^+^, KCl: 2.070 ± 0.031; AP 900: 0.835 ± 0.040/VGAT^+^, KCl: 1.160 ± 0.026; AP 900: 0.823 ± 0.024). These data indicate that while the proportion of synapses that respond to chemical and electrical stimulation remains the same, the number of SV that are released upon chemical depolarization at excitatory and inhibitory synapses is significantly higher in comparison with neurons undergoing electrical field stimulation. This needs to be considered when interpreting the experimental outcomes using both stimulation regimes.

## Conclusion

In this study, we implemented newly developed segmentation algorithm SynEgdeWs in fully automatized frameworks to combine precise, reliable, and fast identification of objects on fluorescently visible and acquired synaptic puncta images with complete pre- and postprocessing. The emerging routines SynEval and ImgSegRout are user-friendly turnkey solutions with the purpose of saving time and reducing human bias.

SynEdgeWs relies on gradient intensity. Since it does not rely on a cutoff intensity threshold to create a binary image, it is less affected by low signal-to-noise ratio or uneven illumination. We have proven SynEdgeWs to outperform algorithms based on threshold application and maxima-guided approaches, as determined by assessment of accurate synapses localization (F1 score) and other measures. Since SynEgdeWs operates iteratively and applies decreasing thresholds for image gradient for each iteration, trade-off between specificity and sensitivity is adjustable depending on image data quality. Due to preservation of shape, this algorithm is potentially suitable to recognize virtually any other cellular structure defined by fluorescent signal that we aim to realize in future routines.

The routines SynEval and ImgSegRout were significantly faster than semi-manual methods. Moreover, the automatic routines are less prone to human error or individual variability, since they hardly involve any steps requiring manual intervention and therefore allow comparison of data obtained by different experimentations or laboratories. Both routines are applicable and adaptable to a wide range of experimental setups. We prepare all software packages for execution in MATLAB runtime enabling the use of software without installing MATLAB and provide routines with a GUI.

The GUI allows specifying further settings such as camera pixel size, magnification, binning, expected diameter of puncta in micrometer to define expected puncta dimensions in pixel counts and to exclude structures out of scope and reasoning. Both routines are equipped with pre- and postprocessing computations partly selectable via the GUI, like bleaching correction in the postprocessing of ImgSegRout or retouching of bright artifact in preprocessing.

Finally, the application of SynEval allowed us to answer a relevant biological question on comparing two different techniques broadly used to induce, monitor, and quantify SV release. Both electrical stimulation and chemical depolarization with KCl have their advantages depending on the experimental system. But without detailed knowledge about their relative potential to evoke SV release, the comparison of experiments using either of them is difficult. In our setting, the proportion of synapses, which are activated, does not differ between both methods. However, a direct comparison revealed that significantly more SV are mobilized upon chemical depolarization compared with electrical stimulations. We conclude that both electrical stimulation and chemical depolarization merit their place in different experimental settings, but chemical depolarization tends to mobilize vesicles that are not releasable upon intense electrical stimulation. It will be interesting to approach the molecular determinants of the observed difference in future experiments.

## Resource Identification Initiative

All catalog numbers and RRID used in the study are given in Supplementary Material Table S3, S4.

## Data Availability

The datasets presented in this study can be found in online repositories. Generated code and datasets for this study can be found here: https://github.com/EvaMWe/Synapse-quantification.
