# Supplementary material for: Development and Application of Automatized Routines for Optical Analysis of Synaptic Activity Evoked by Chemical and Electrical Stimulation
Source: Front Bioinform. 2022 Feb 15;2:814081. doi: 10.3389/fbinf.2022.814081 (PMC9580924; doi:10.3389/fbinf.2022.814081)
Supplement: Supplementary file 1 [file DataSheet1.PDF]

Supplementary Material

1 Supplementary Methods

1.1 Overview of processes implemented in preprocessing

Basic functions: Background subtraction and normalization:

- Creation of a kernel K:
  $K^w(i,j) = \frac{1}{n}\sqrt{i^2 + j^2}$ with w = 21 , n =sum(K)
- Convolution between image and kernel: blurred image = background image
- Subtraction of blurred image from original image
- Set values < 0 to 0
- Normalization of all pixels as $I\text{-}I_{min}/(I_{max}\text{-}I_{min})$

Optional processing steps: selectable by user

Denoising:

- Establishing of a Gaussian filter (w = 3)
- Convolution of filter and image

Retouch bright regions:

- Definition of a structure element SE, disc shaped, radius 21;
  // get threshold (compare Fig. S1)
- Vectorising image values
- Creation of histogram: binData (counts in bins) and intensities (edges)
- Inverting bins: binDataInv
- Cumulative sum of binDataInv (CS)
- Dividing CS by cumulative number of bins (CSL estimates 1st derivative) and calculating differences between adjacent elements to get approximate derivative (CSLdiff)
- Condition: CSLdiff >= -0.5 (plateau) → location to set threshold// coming from high intensity values → get first value fulfilling condition
- Sbright: threshold with transferred index to original binData
- Extraction of a section from CSL to calculate second threshold (coming from dark side) (CSL (end):-1:Sbright)
- Calculating of differences between adjacent elements and application of condition (tracediff >= -0.5) → get first index fulfilling this condition
- Sdark = threshold with transferred index to original binData
- Location S: (Sbright + Sdark)/2
- Get intensity value at S as threshold IntS
  // apply threshold on image and subtract background
- Creation of mask (logical FALSE) with size of image
- Replace logical FALSE to TRUE at positions in mask, where corresponding image pixel value > IntS
- Dilation with SE

- Replacement of image values with background value according to mask (calculated prior as background image)

1.2 Iterative thresholding in SynEdgeEval

- Setting of number of iterations by user in graphical user interface GUI
- Convolution of the image with Sobel operator in MATLAB, returning an approximation of derivatives for horizontal G_x and vertical G_y changes.
- Calculation of image gradient as:

$$image\ gradient = \sqrt{G_x^2 + G_y^2}$$

- Setting the threshold as the gradient value at the point in the histogram with a defined fraction (pth) of pixels having higher values [counts, bins]

```
k = 0;
while sum(counts(end-k:end))< pth*sum(counts)
    k = k+1;
end
h=bins(end-k+1);
```

- Increase of pth with each iteration (x = step-1), resulting in a decrease in gradient threshold: $pth = \alpha \cdot 1.259^x$, default $\alpha = 0.005$
- Dilation, flood filling, watershed transformation and refinement
- Exclusion of segmented regions for the next iteration
- Clearance of overlaps and artefacts due to iterative morphological operations by calculating labelled matrix and discarding artefacts

1.3 Benchmarking of SynEdgeEval

https://github.com/EvaMWe/Synapse-Detection contains all custom-coded tools used for benchmarking

Brief description:

(1) Cut-off thresholding to create binary image (Thr)
  - Limitations of puncta size, here: minarea = 6 pixels; maxarea = 200 pixels (13µm camera pixel size, 60x magnification, no binning);
  - Determination of counter pth to determine a threshold by means of the intensity histogram [counts, bins]: pth defines the position in the histogram with a defined fraction (pth) of higher values;
  - Creation of intensity histogram [counts, bins] and finding the position fulfilling the condition:

```
k = 0;
while sum(counts(end-k:end))< pth*sum(counts)
    k = k+1;
end
h=bins(end-k+1);
```

  - Get corresponding intensity value as threshold: threshold = bins(end-k+1)
  - Creation of binary image by thresholding
  - Flood filling to fill wholes (in-built MATLAB: imfill)

- Clear borders
- Get regions (in-built MATLAB function: regionprops)
- Application of maximum and minimum pixel number for puncta to exclude regions out of expected size scope
- Creation of a new binary image

(2) Thresholding combined with marker-controlled watershed transformation (ThrWs)
  - First part: same procedure as (1)
  - Watershed transformation (getCentroids, getSingles, precising)
  - Creation of binary image

(3) Local maximum controlled by global threshold (locMax) according to:
According to (Sbalzarini & Koumoutsakos, 2005)
  - Radius of the mask used in dilation to get centroids: w = 3 [in pixels]
  - Size of the circle radius marking synaptic puncta: radius = 3 [in pixels]
  - Percentage of histogram counts, coming from high values: pth = 5

(4) SynEdgeWs: the factor α (s. 1.2) was adjusted to 0.06 due to cropping of the images

1.4 Measures to quantify segmentation quality

To evaluate the performance of the segmentation algorithms, we compared their segmentation results to the ground truth segmentation data that we obtained by manual segmentation. Therefore, we used the measures we termed in the manuscript F1 score and dice coefficient. Both measures rely on following equation:

$$F1 = \frac{2 \cdot precicion \cdot recall}{precision + recal} = \frac{TP}{TP + 0.5(FP + FN)}$$

with
TP = true positive
FP = false positive
FN = false negative

Originating from the equal formula, the measures differ in their definition of TP. For the measure, that we termed F1 score in the manuscript, we determined TP by comparing segmented regions individually to their potential match on GT. For that, we compared coordinates from individual ROI with the coordinates of ROI on GT data. We used intersection over union to infer a true positive (TP) and set TP, when there is an overlap of ground truth (GT) and a detected ROI. In contrast, the measure that we termed dice coefficient (to discriminate it from the previous explained F1 score) bases on the comparison of the binary segmentation masks in a pixel-wise manner. Here, the binary segmentation mask, obtained by an algorithm, is compared to GT segmentation mask. According to the MATLAB documentation (The MathWorks, 2017) and transferred to our benchmarking process, the corresponding formula compares the set of segmented pixels on GT (A) with the set of segmented pixels with an algorithm (B):

$$dice(A,B) = \frac{2 \cdot |intersection(A,B)|}{|A| + |B|}$$

In addition, we used a MATLAB built-in function to calculate the boundary F1 score (bf score). According to the MATLAB-documentation (The MathWorks, 2017), the calculation of bf score bases, like the previously described measures F1 score and dice coefficient, on following formula:

$$bf\ score\ = \frac{2 \cdot precicion \cdot recall}{precision + r}$$

In contrast, it measures how close the predicted boundary of an object matches the GT boundary (Csurka *et al*, 2004).

1.5 Steps for semi-manual analysis to benchmark running time of SynEval

1. Loading of images in Fiji
2. Subtraction of background (Process > Subtract background > Rolling ball radius: 50.0 pixels)
3. Determination of threshold for channel 2 and channel 3 images (Image > Adjust > Threshold) → saving of values
4. Segmentation on channel 1 (Plugins > SynQuantVid > Z-Score: 10.00, Min Particle Size: 6 Max Particle Size: 200
5. Measure of the mean fluorescence intensity (MFI) on channel 1 and saving the data as a MS Excel Sheet (popped up ROI-Manager > Measure)
6. Application of segmentation mask (apply from ROI-Manager) on channel 2 and channel 3 images, measurement of MFI and saving the data as a MS Excel Sheet.
7. Further calculations, including classification of puncta using information from channel 2 and channel 3 by threshold application.

2 Supplementary Tables

TableS1: Settings for calculation of puncta size range

Setting	default	unit
Camera pixel size	13	µm
Magnification (objective)	60	
binning	1	
Minimum diameter	0.5	µm
Maximum diameter	3	µm

TableS2: Overview of features returned by SynEval

Feature	Description
Number of puncta	Number of detected synaptic puncta on channel-1 image
Fraction channel-2	Fraction of detected synaptic puncta simultaneously positive for marker channel-2: $\frac{detected\ in\ channel-1\ \&\ positive\ in\ channel-2}{detected\ in\ channel-1}$
MFI channel-2	Mean fluorescence intensity of synaptic puncta positive in channel-2 and detected in channel-1
Number of puncta (channel-3)	Number of detected synaptic puncta positive in channel-3 and detected in channel-1
Fraction channel-3	Fraction of detected synaptic puncta simultaneously positive for marker channel-3: $\frac{detected\ in\ channel-1\ \&\ positive\ in\ channel-3)}{detected\ in\ channel-1}$
MFI channel-3	Mean fluorescence intensity of synaptic puncta positive in channel-3 and detected in channel-1
Fraction channel-2 in (channel-3 & channel -1)	Fraction of detected synaptic puncta simultaneously positive in channel-2 & positive in channel-3: $\frac{positive\ in\ channel-2\ \&\ pos.channel-3\ \&\ detected\ in\ channel-1}{detected\ in\ channel-1\ \&\ positive\ in\ channel-3}$
MFI channel-2 in (channel-3 & channel-1)	Mean fluorescence intensity of synaptic puncta positive in channel-3 and positive in channel -2 and detected in channel-1

TableS3: Overview of antibodies, reagents and equipment

Antibodies	Supplier	Cat. Number
anti guinea pig Cy 5	Jackson IR	#706-175-148
anti rabbit Alexa 488	Jackson IR	#711-545-152
guinea pig anti synapsin 1,2	Synaptic Systems	#106004
mouse anti synaptotagmin1Oyster 550	Synaptic Systems	#105311C3
rabbit anti VGAT	Synaptic Systems	#131003
rabbit anti VGLUT1	Synaptic Systems	#135303
**Cell culture media and reagents**	**Supplier**	**Cat. Number**
antibiotics/antimycotics	Thermo Fisher	#15240062
APV	Tocris Bioscience	#0106
B-27™ supplement	Gibco	#17504-044
bafilomycin A1	Calbiochem	#88899552
CNQX	Tocris Bioscience	#0190
DMEM	Thermo Fisher	#41966029
fetal calf serum	Biochrom	#S0015
L-glutamine	Thermo Fisher	#25030024
poly-L-lysine	Sigma Aldrich	#P1524
**Cloning reagents and Plasmids**	**Supplier**	**Cat. Number**
BamHI-HF®	NEB	#R3136S
EcoRI-HF®	NEB	#R3101S
NEBuilder® HiFi DNA Assembly	NEB	#2621
psPAX2	Addgene	#12260
pVSVG	Addgene	#138479
SypmOr	Egashira et al., 2015	
**Equipment**	**Supplier**	**Cat. Number**
glas coverslips	VWR	#6311342
imaging chamber	Warner Instrument	#RC-49MFSH

TableS4: Microscope setup

Microscope setup	Supplier
Nikon Eclipse Ti epifluorescence microscope, with automated perfect focus system and	Nikon Corporation

60X/NA1.2 water-immersion objective (CFI Plan Apo VC)	
A 385 stimulus isolator	World Precision Instruments, Sarasota, Florida, USA
STG-4008 stimulus generator	Multi Channel Systems, Reutlingen, Germany
Led-HUB lamp	Omicron-laserage Laserprodukte GmbH, Rodgau, Germany
iXon EM + 885 EMCCD Andor camera	Andor Technology

TableS5: Data from time running experiment of SynEval (one iteration)

Experiment	Time per run [sec]	Nb of analyzed sections	Mean time per section [sec]
Exp1-Cov1	346	10	34.6
Exp1_Cov2	323	11	29.3
Exp1_Cov3	450	11	40.9
Exp1_Cov7	318	8	39.8
Exp1_Cov8	401	12	33.4
Exp1_Cov9	384	10	38.4
Exp1_Cov10	357	11	32.5
Exp2_Cov1	400	10	40
Exp2_Cov2	483	11	43.9
Exp2_Cov3	401	10	40.1
Exp2_Cov4	368	10	36.8
Exp2_Cov7	446	11	40.5
Exp2_Cov8	439	10	43.9
Exp2_Cov9	491	11	44.6
Exp2_Cov10	475	10	47.5
**→ 39.09 ± 1.25 (4.85) [sec]**			

TableS6: Data from time running experiment of ImgSegRout

Run	Nb of analyzed image stacks per run	time [sec]
1	5	131
2	4	106
3	3	119

→Mean: 29.67 ±2.09 (6.28) [sec]

3 Supplementary Figures

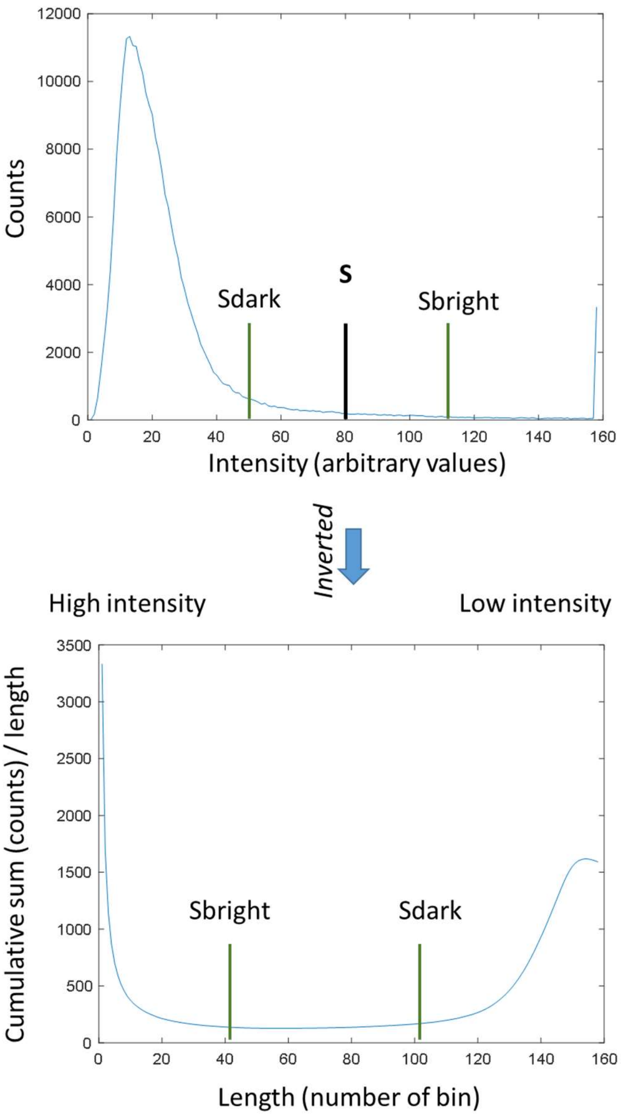

Figure S1: Calculation of threshold for retouching bright regions within preprocessing (A) Intensity histogram. (B) Two thresholds are set in CMS graph (CMS: cumulative sum of counts from the inverted intensity histogram divided by length (number of bins)). Thresholds are set at positions where the slope becomes close to zero. Coming from the left side (high intensities) the threshold is indicated as Sbright and coming from the right side (low intensities) the threshold is indicated as Sdark. To get the final threshold, the mean value between Sbright and Sdark is calculated (S). The index of S is converted to the original intensity histogram (A) and the belonging intensity value is determined.

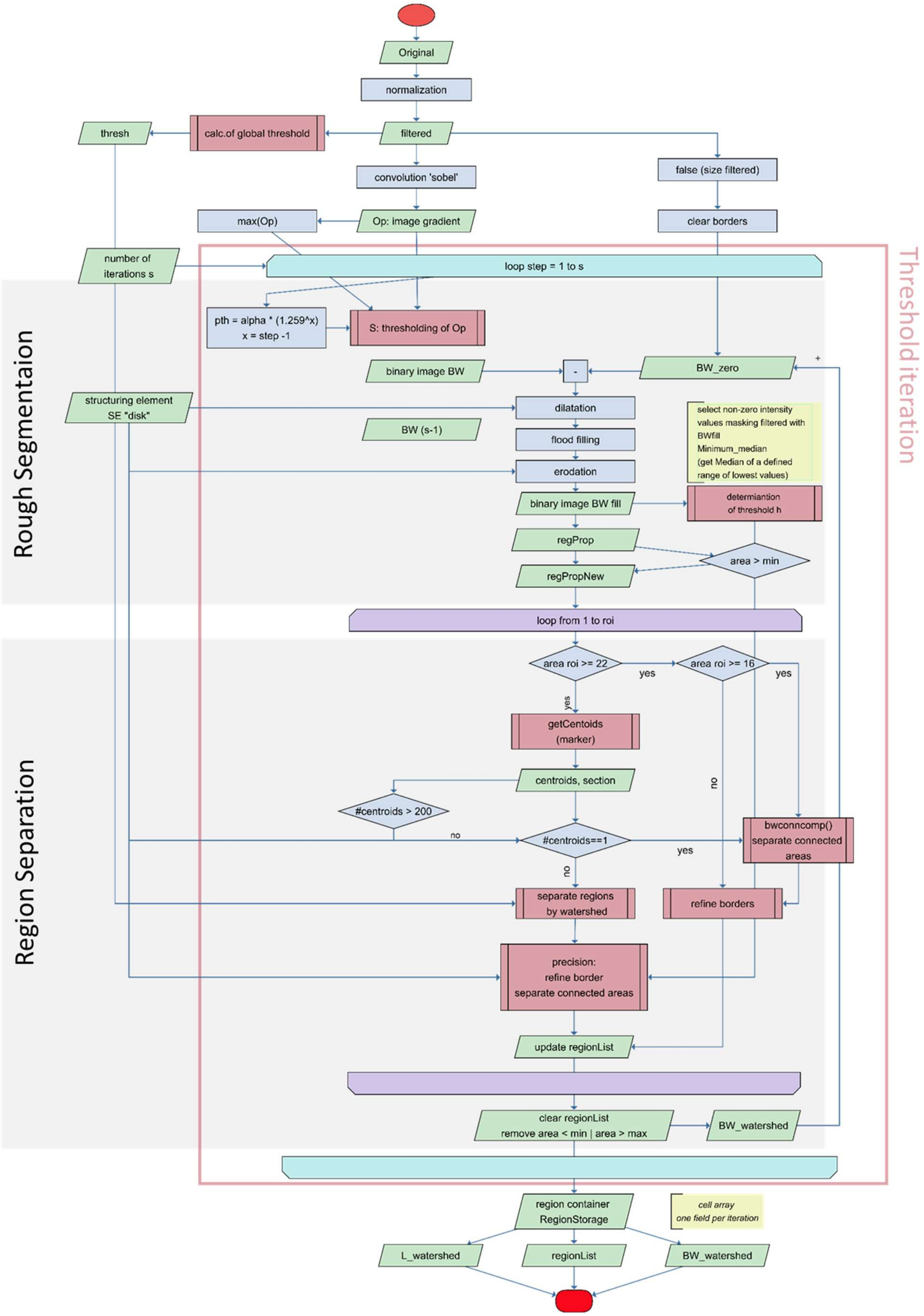
Original
normalization
thresh
calc.of global threshold
filtered
convolution 'sobel'
false (size filtered)
max(Op)
Op: image gradient
clear borders
number of iterations s
loop step = 1 to s
Threshold iteration
Rough Segmentaion
pth = alpha * (1.259^x)
x = step -1
S: thresholding of Op
binary image BW
-
BW_zero
+
structuring element SE "disk"
dilatation
BW (s-1)
flood filling
select non-zero intensity values masking filtered with BWfill
Minimum_median
(get Median of a defined range of lowest values)
erodation
binary image BW fill
determiantion of threshold h
regProp
area > min
regPropNew
loop from 1 to roi
Region Separation
area roi >= 22
yes
area roi >= 16
yes
yes
getCentoids (marker)
centroids, section
no
#centroids > 200
no
#centroids==1
yes
bwconncomp()
separate connected areas
no
separate regions by watershed
refine borders
precision:
refine border
separate connected areas
update regionList
clear regionList
remove area < min | area > max
BW_watershed
region container
RegionStorage
cell array
one field per iteration
L_watershed
regionList
BW_watershed

Figure S2: Flow chart of SynEdgeWs.

The schema of SynEdgeWs is illustrated. The outer loop (turquoise) indicates the threshold iteration. For each loop, threshold application generates a binary image from the image gradient (filtered). Rough regimentation is completed by the application of several morphological operators (MATLAB in-built functions dilation, imfill, erosion). After the clearance of regions going below the minimum number of pixels, a list of ROI coordinates is generated (regPropNew). Connected regions are separated by application of a marker-controlled watershed transformation (region separation). Centroids for each section (roi) are detected as markers (getCentroids). Regions with a pixel size below a threshold are excluded from watershed transformation. All regions are checked for artefacts (clear regions), that might arise due to morphological operators, borders are refined (refine borders) and MATLAB in-built function bwconncomp() separates components that are still connected. A region list is stored for each iteration in RegionStorage and is merged in a final step resulting in the final region list (regionList), binary image (BW_watershed) and labelled matrix (L_watershed)

.

Csurka G, Larlus D, Perronnin F, Meylan F (2004) What is a good evaluation measure for semantic segmentation. *IEEE PAMI* 26

Sbalzarini IF, Koumoutsakos P (2005) Feature point tracking and trajectory analysis for video imaging in cell biology. *J Struct Biol* 151: 182-195

The MathWorks I, 2017. dice.

The MathWorks I, 2017 bfscore.
